# Unravelling the complex drug–drug interactions of the cardiovascular drugs, verapamil and digoxin, with P-glycoprotein

**DOI:** 10.1042/BSR20150317

**Published:** 2016-03-16

**Authors:** Kaitlyn V. Ledwitch, Robert W. Barnes, Arthur G. Roberts

**Affiliations:** * Department of Pharmaceutical and Biomedical Sciences, University of Georgia, Athens, GA 30602, U.S.A.

**Keywords:** ABC transporter, cardiovascular, drug transport, fluorescence, NMR

## Abstract

Drug–drug interactions (DDIs) and associated toxicity from cardiovascular drugs represents a major problem for effective co-administration of cardiovascular therapeutics. A significant amount of drug toxicity from DDIs occurs because of drug interactions and multiple cardiovascular drug binding to the efflux transporter P-glycoprotein (Pgp), which is particularly problematic for cardiovascular drugs because of their relatively low therapeutic indexes. The calcium channel antagonist, verapamil and the cardiac glycoside, digoxin, exhibit DDIs with Pgp through non-competitive inhibition of digoxin transport, which leads to elevated digoxin plasma concentrations and digoxin toxicity. In the present study, verapamil-induced ATPase activation kinetics were biphasic implying at least two verapamil-binding sites on Pgp, whereas monophasic digoxin activation of Pgp-coupled ATPase kinetics suggested a single digoxin-binding site. Using intrinsic protein fluorescence and the saturation transfer double difference (STDD) NMR techniques to probe drug–Pgp interactions, verapamil was found to have little effect on digoxin–Pgp interactions at low concentrations of verapamil, which is consistent with simultaneous binding of the drugs and non-competitive inhibition. Higher concentrations of verapamil caused significant disruption of digoxin–Pgp interactions that suggested overlapping and competing drug-binding sites. These interactions correlated to drug-induced conformational changes deduced from acrylamide quenching of Pgp tryptophan fluorescence. Also, Pgp-coupled ATPase activity kinetics measured with a range of verapamil and digoxin concentrations fit well to a DDI model encompassing non-competitive and competitive inhibition of digoxin by verapamil. The results and previous transport studies were combined into a comprehensive model of verapamil–digoxin DDIs encompassing drug binding, ATP hydrolysis, transport and conformational changes.

## Abstract (precis)

P-glycoprotein (Pgp) plays a major role in promoting drug–drug interactions (DDIs) with verapamil and digoxin. In the present study, we present a comprehensive molecular and mechanistic model of Pgp DDIs encompassing drug binding, ATP hydrolysis, transport and conformational changes.

## INTRODUCTION

Drug–drug interactions (DDIs) involving cardiovascular therapeutics and their related toxicity continue to represent serious challenges to effective treatment of patients with heart disease [1–5]. In one previous [5] study, DDIs from co-administration of cardiovascular drugs were implicated in ∼50% of adverse drug reactions in patients receiving therapy. The P-glycoprotein (Pgp) transporter is an ATP-powered efflux pump that plays a major role in cardiovascular DDIs and effluxes a diverse range of cardiovascular therapeutics [6,7]. The transporter is expressed in the brain, intestines, liver, placenta and the kidneys [8,9] and at relatively low levels in the heart [10]. The expression level is also influenced by genetic polymorphisms and cardiomyopathy [11,12].

DDIs with the transporter occur because many cardiovascular drugs are substrates for and functional inhibitors of the transporter [4,7,13,14]. This is particularly problematic for cardiovascular drugs with relatively low therapeutic indexes such as antiarrhythmic drugs and oral anticoagulants because co-administration with these drugs can lead to elevated drug plasma concentrations and increased toxicity [7].

**Figure 1:**
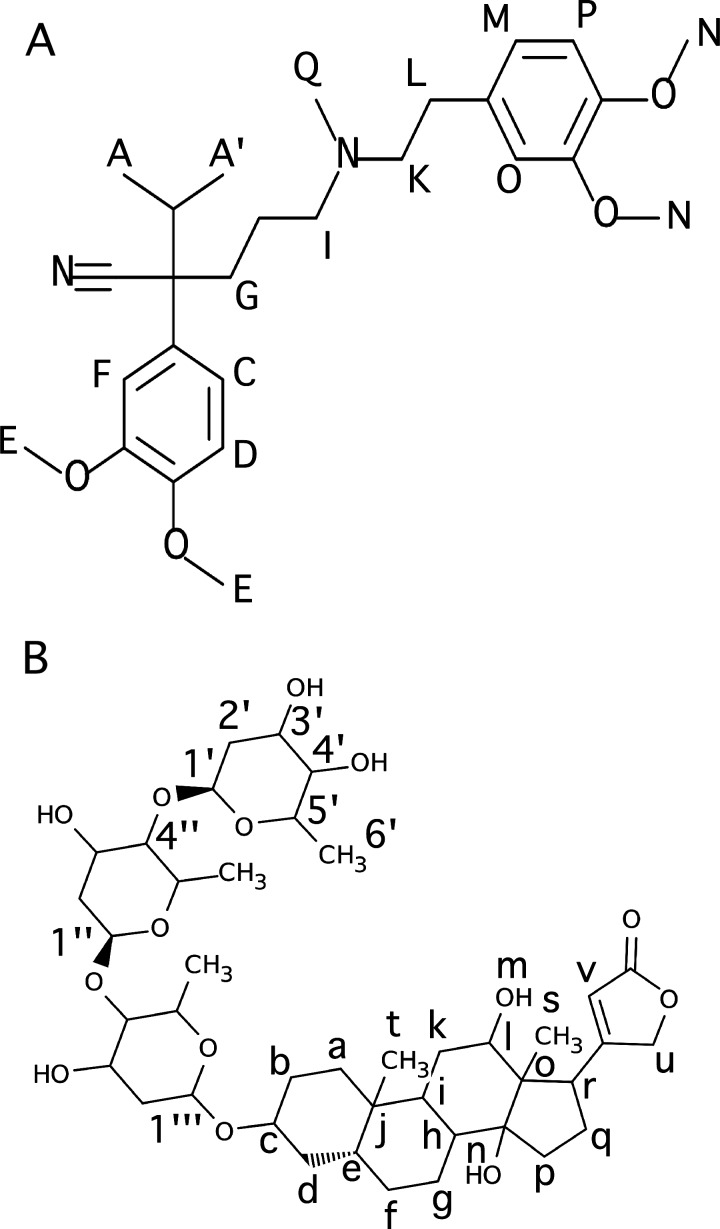
Molecular structures of (**A**) verapamil and (**B**) digoxin with the nuclei labelled

The calcium channel blocker verapamil (Figure 1A), which is commonly used to control hypertension, chest pain and arrhythmia [15–19], functions as a substrate and an inhibitor of the transporter [7]. From results of *in vitro* studies, the drug is known to activate Pgp-coupled ATP-hydrolysis [20]. This drug manifests a spectrum of characteristics, ranging from being a good substrate to a non-substrate for the transporter, which depends on the cell type being evaluated in *in vitro* cell studies [21–26] or host tissue type in *in vivo* studies [27–29]. Although the actual molecular details of these interactions are currently unknown, the drug has been shown to inhibit the ATPase activity of a second drug by competitive, non-competitive and allosteric mechanisms in an *in vitro* study [30]. Verapamil has also been shown to inhibit cardiovascular drug transport by human Pgp *in vivo* [4,31,32].

The cardiac glycoside digoxin (Figure 1B), which has a relatively low therapeutic index, is widely used to treat atrial fibrillation and heart failure [33]. The drug is primarily excreted by the Pgp transporter in the kidneys [34,35]. Importantly, this drug is often co-administered with verapamil, which is known to non-competitively inhibit human Pgp-mediated digoxin transport based upon *in vitro* studies [36,37]. These findings strongly suggest that both drugs are simultaneously bound to the transporter. Inhibition of human Pgp transport by verapamil *in vivo* is known to decrease the extent of renal tubular elimination of digoxin. This finding correlated with increased digoxin blood plasma concentrations from 60 to 90% [32,36] and lead to adverse drug reactions from digoxin toxicity [31,38].

Because verapamil and digoxin have been the focus of a number of *in vitro* [20] and *in vivo* studies [27], these drugs are ideal for studying DDIs with the transporter. Many molecular and mechanistic details of verapamil–digoxin DDIs with Pgp remain unresolved. This information is essential for defining a general DDI mechanism, for identifying therapeutics that have a high probability of exhibiting DDIs with Pgp and for ameliorating DDIs from commercially available therapeutics with Pgp.

The effect of verapamil and digoxin on the Pgp-coupled ATPase activity, the interactions of verapamil and digoxin with Pgp and the effect of verapamil and digoxin on Pgp conformation were investigated with Pgp reconstituted into liposomes. The drug-induced ATPase activation kinetics of Pgp in the presence of verapamil and digoxin allowed us to estimate the minimum number of drug-binding sites. To explore the effect of verapamil on the affinity of digoxin, digoxin's affinity to Pgp in the presence of several verapamil concentrations was estimated using intrinsic protein fluorescence. The molecular interactions between the drugs and Pgp were investigated by the saturation transfer double difference (STDD) NMR technique. Drug-induced effects on Pgp conformation were studied by acrylamide quenching of tryptophan fluorescence. Additionally, Pgp-coupled ATPase activity kinetics were measured with a panel of verapamil and digoxin concentrations, and fit to a DDI model of drug-induced ATPase activation. This information was combined with previous transport studies to produce a comprehensive mechanistic and molecular model of verapamil–digoxin DDIs.

## EXPERIMENTAL

### Materials

Verapamil hydrochloride was purchased from Fagron. Digoxin, ethylene glycol tetraacetic acid (EGTA) and imidazole were purchased from Alfa Aesar. The detergent used in protein purification, *n*-dodecyl-β*-*D-maltoside (DDM), was purchased from EMD Millipore Corporation. *Escherichia coli* total lipid extract powder was purchased from Avanti Polar Lipids Inc. DTT was purchased from Gold Biotechnology. Deuterium oxide (^2^H_2_O) was purchased from Cambridge Isotope Laboratories. The remaining chemicals were purchased from Sigma–Aldrich.

### Expression and purification of the mouse Pgp transporter

The wild-type His-tagged mouse Pgp transporter was purified from *Pichia pastoris* as described with some modifications [39,40]. The yeast cells were grown and induced with methanol at the Bioexpression and Fermentation Facility at the University of Georgia in a 32 l DCI-Biolafitte fermenter with a 20 l working volume using a similar strategy as [40]. Instead of using glass bead breaking or the French press to crack the yeast cells [39,40], the cells were cracked by a minimum of six passes by liquid nitrogen freezing and blending [41]. To reduce the amount of DDM in our activity assays and during liposome preparation, no additional DDM was added after the nickel-nitrilotriacetic acid (Ni-NTA) column step. Typical protein purification yields were 12±2 mg for 100 g of wet weight cells, which is similar to previous yields [39]. SDS/PAGE analysis of the protein showed that it was >95% pure. The protein was concentrated up to 150 μM in Amicon Ultra-15 100 kDa cut-off filters (EMD Millipore, Billerica, MA) and stored at −80°C in 10 mM Tris/HCl, 30% glycerol, pH 8.0. The concentration of detergent-solubilized Pgp was measured using the DC Protein Assay Kit II (Bio-Rad Laboratories) or using the molar absorption coefficient of 1.28 ml·mg^−1^·cm^−1^ (0.181 μM^−1^·cm^−1^) [39].

### Reconstitution of Pgp into liposomes

Pgp was reconstituted into 400 nm unilamellar liposomes using the filter extrusion method [42,43]. The liposomes were composed of 80% w/v Avanti *E. coli* Total Lipid Extract (Avanti Polar Lipids) with a defined lipid profile and 20% w/v cholesterol. Lipids and cholesterol were mixed together in chloroform to a final volume and concentration of 10 ml and 10 mg·ml^−1^ respectively. This organic solution was evaporated to dryness in a Buchi Rotavapor Model R-114 (Buchi). This was resuspended in 10 ml of 0.1 mM EGTA and 50 mM Tris/HCl (pH 7.4). The suspension was freeze thawed at least 10 times using liquid nitrogen. The rehydrated lipid was put through a LIPEX extruder 11 times (Northern Lipids) with a 400 nm cutoff Millipore filter (EMD Millipore). Approximately 100 μM of Pgp was dialysed against HEPES buffer (20 mM HEPES, 100 mM sodium chloride, 5 mM magnesium chloride, 2 mM DTT, pH 7.4) for 2 h to remove residual detergent. Then 50 μM of dialysed protein and 4 mg·ml^−1^ liposomes were incubated for 1 h. This was then dialysed for another 2 h against HEPES buffer to promote integration of the protein into the liposomes. To remove aggregated Pgp, the reconstituted liposomes were centrifuged for 5 min at 100 ***g*** in a Sorvall Legend Micro 21 centrifuge (ThermoScientific). To determine the orientation of mouse Pgp in the liposomes, the permeability of the reconstituted liposomes was tested with CHAPS detergent to expose nucleotide-binding domains (NBDs) oriented within the liposome [20,44,45]. Since there was no increase in the ATPase activity with increasing CHAPS concentrations, Pgp was assumed to be in an inside-out orientation.

### ATPase activity measurements

The ATPase activity of the Pgp transporter was measured using the Chifflet method [46]. The method estimates the ATPase activity by measuring the concentration of free P_i_ after ATP hydrolysis through the formation of a P_i_–molybdenum complex, which produces a strong absorbance signal at 850 nm. The absorbance at 850 nm was measured on a 96-well plate in a FlexStation 3 spectrometer (Molecular Devices). The ATPase activity of verapamil and digoxin was measured with 50 nM Pgp in Chifflet buffer (150 mM ammonium chloride, 5 mM magnesium sulfate, 0.02% w/v sodium azide, 50 mM Tris/HCl, 2 mM DTT, pH 7.4).

Traditionally, simple enzyme kinetics have been analysed using linear transformations such as the Lineweaver–Burk (double reciprocal), Hans–Woolf or Eadie–Hofstee plots [47,48]. However, these plots suffer from a lack of variable independence across the axes and biasing of the error and the data points [49–51]. These methods have generally been superseded by non-linear regression methods that are significantly more accurate and no longer computationally inaccessible [50]. Therefore, for ATP hydrolysis kinetics that were monophasic, the ATP hydrolysis rate (*v*), the maximum ATP hydrolysis rate (*V*_MAX_), the basal ATPase hydrolysis rate (*v*_basal_) and the Michaelis–Menten constant (*K*_m_) were estimated with the Michaelis–Menten equation (eqn 1) [48,52]:

1 $$v\;=\frac{V_{\mathrm{MAX}\;}\left[L\right]}{K_{m}+\left[L\right]}\;+v_{\mathrm{basal}}$$

For ATP hydrolysis kinetic curves showing biphasic substrate inhibition, the *V*_MAX_, *K*_m_ and the inhibitory constant (*K*_i_) were estimated with eqn (2) [48,52]:

2 $$v\;=\frac{V_{\mathrm{MAX}}\;}{1+\frac{K_{m}}{\left[L\right]}+\;\frac{\left[L\right]}{K_{i}}}\;+v_{\mathrm{basal}}$$

For more complicated kinetics, fitting equations have been developed in some cases, but may require specialized numerical methods to solve and may result in multiple solutions [53].

To overcome these challenges, a variety of advanced software modelling packages have been developed to fit arbitrary kinetic models including the free complex pathway simulator (COPASI) [54] and the proprietary Berkeley Madonna (University of California, Berkeley, CA). For ATP hydrolysis kinetics observed in the presence of both verapamil and digoxin, the ATPase activity curves were fit to kinetic models using the evolutionary programming fitting algorithm in the COPASI software [54].

### Fluorescence spectroscopy

Quenching of intrinsic protein fluorescence has been used to measure the binding affinity of a chemically-diverse range of ligands with Pgp [55,56]. Drug-induced quenching of protein fluorescence with Pgp reconstituted in liposomes was investigated on an Olis DM 45 spectrofluorimeter (Olis Corp). All fluorescence samples contained 1 μM liposome-reconstituted Pgp in Chifflet buffer (pH 7.4). Fluorescence emission was measured at 333 nm following excitation between 260 and 295 nm to minimize inner filter effects and background fluorescence. Drug-induced fluorescence quenching was corrected (*F*_corrected_) for background fluorescence, dilution and inner filter effects with eqn (3) [57]:

3 $$F_{\mathrm{corrected}}=\;\left(F-B\right)10^{\frac{\left({ɛ}_{\mathrm{ex}\;}b_{\mathrm{ex}\;}+\;{ɛ}_{\mathrm{em}}b_{\mathrm{em}}\right)\left[Q\right]}{2}}$$

where *F* is the measured protein fluorescence, *B* is the background and [*Q*] is the quenching ligand concentration. The molar absorption coefficients (ε) for excitation and emission are ε_ex_ and ε_em_ respectively. Verapamil was transparent above 300 nm and had ε_280 nm_ and ε_295 nm_ of 4 and 0.27 mM^−1^·cm^−1^ respectively. Digoxin was transparent above 250 nm. The pathlength (*b*) along the excitation and emission axes are *b*_ex_ and *b*_em_ respectively. Drug-induced quenching of protein fluorescence from complexation of the ligand to the protein is known as static quenching, and can be used to estimate the drug's affinity. Drug-induced fluorescence quenching from random collisions with the protein is known as dynamic quenching [57]. Regardless of the nature of the quenching, the fluorescence quenching curves were fit to eqn (4) [57]:

4 $$F_{\mathrm{corrected}}=\;\frac{F_{\mathrm{corrected},0}}{1+K\left[Q\right]}$$

where *F*_corrected,0_ is the protein fluorescence in the absence of a quenching ligand and *K* is the association constant (*K*_A_) or the Stern–Volmer quenching constant (*K*_SV_) in the case of a static and dynamic quenching processes respectively. The two different quenching mechanisms can be differentiated by measuring the protein's fluorescence life time in the presence of the quenching ligand or by performing the fluorescence titration experiments at two different temperatures [57]. In the latter case, the *K* will increase with increasing temperature for dynamic quenching by increasing the collisional frequency of the quencher and will decrease in the case of static quenching by decreasing the residence time of the quenching ligand.

Acrylamide is a neutral aqueous collisional quencher that has been widely used to probe the accessibility of tryptophans in proteins and probe changes in tertiary structure [55,58]. Dynamic quenching of intrinsic tryptophan fluorescence by acrylamide has been used to probe conformational changes of Pgp [55,58,59]. For these experiments, fluorescence emission with Pgp reconstituted in liposomes was measured at 333 nm following excitation at 295 nm. Control acrylamide titrations were performed in the presence of *N*-acetyl-L-tryptophanamide (NATA) to estimate the degree of non-specific quenching [55]. Fluorescence intensities were corrected for with eqn (3) [57]. To produce the Stern–Volmer plots, the *F*_corrected,0_/*F*_corrected_ was plotted as a function of the acrylamide concentration. The degree of dynamic tryptophan quenching was estimated from the slopes of the Stern–Volmer curves, which is related to *K*_SV_ by *F*_corrected,0_/*F*_corrected_ *=* 1 + *K*_SV_[*Q*] [57].

### NMR

All NMR experiments with verapamil and digoxin ^1^H NMR spectra were performed on a 600 MHz Varian INOVA spectrometer at 25°C equipped with a 5 mm z-gradient ^1^H{^13^C/^15^N} cryoprobe. The ^1^H NMR peaks were assigned using standard ^1^H 1D and 2D NMR techniques. NMR spectra were analysed using the iNMR software (http://www.inmr.net) and Igor Pro 6.2 (Wavemetrics). The ^1^H NMR peak assignments for verapamil and digoxin are shown in Supplementary Figure S1 in the Supplementary Information and were essentially identical with previous ^1^H NMR peak assignments [60,61].

The saturation transfer difference (STD) NMR technique is a well-established method for probing ligand–protein interactions [62]. With this technique, the protein is selectively excited at a frequency outside of the ligand ^1^H NMR peaks, the saturation is transferred from the excited protein to the ligand through spin diffusion and the ligand STD NMR signal is observed [63]. However, in the case of liposomes reconstituted with Pgp, there will be significant interference because of saturation transfer between the liposome membrane and the drugs. This interference can be subtracted from the saturation transfer between the drug and the protein by the NMR technique called STDD [64–67]. The STDD NMR procedure for membrane proteins was performed as described in [68]. STDD NMR samples contained 1 μM Pgp reconstituted into liposomes in 100 mM potassium phosphate buffer [80% ^2^H_2_O (99.9%) and 20% ddH_2_O, pH 7.4]. The STDD NMR experiments were performed with a double pulsed field gradient spin echo pulse sequence to suppress background water, a 2s train of 50 ms shaped saturation pulses to selectively excite the protein and a total relaxation delay of 5 s [63]. The number of transients collected for the on resonance and the off resonance spectra were 512. To minimize saturation transfer between the drugs and the liposomes, samples were selectively irradiated at a frequency of 10.5 ppm. Control experiments were performed under identical conditions with liposomes and the drugs. To produce the STDD NMR spectrum, ^1^H STD NMR spectrum of liposomes with drugs was subtracted from the ^1^H STD NMR spectrum of Pgp reconstituted in liposomes with drugs (Δ*I*). The STDD NMR subtraction procedure is demonstrated with verapamil in Supplementary Figure S2 in the Supplementary Information. The STDD amplification factor was calculated using the following equation based on the STD amplification factor (eqn 5) [63]:

5 $$\mathrm{STDD}\;\mathrm{amplification}\;\mathrm{factor}\;=\;\frac{\left[L\right]}{\left[P\right]}\frac{\Delta I}{I_{0}}$$

where [*P*] is the protein concentration and *I*_0_ is the amplitude of the ^1^H NMR peaks in the absence of saturating pulses.

## RESULTS

### The effect of verapamil and digoxin on the Pgp-coupled ATPase activity

**Figure 2:**
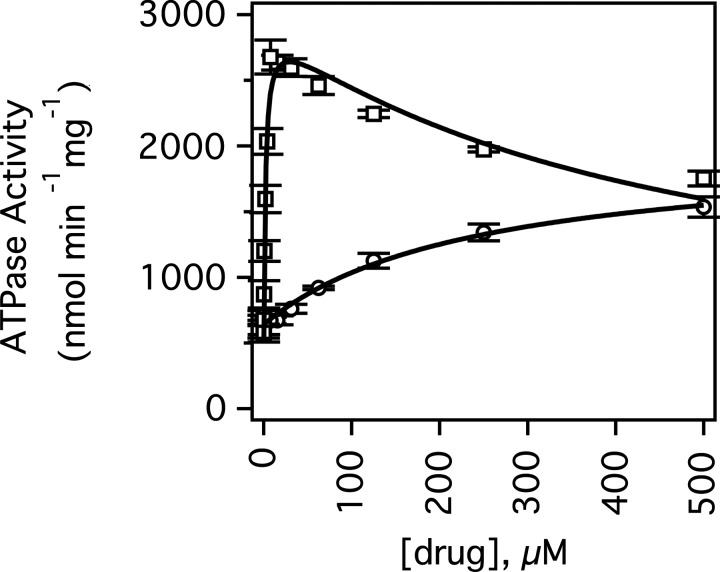
Verapamil and digoxin-induced ATPase activation of Pgp The Pgp-coupled ATPase activity as a function of verapamil (open squares) and digoxin (open circles) concentrations. The fits are shown as solid lines. Error bars represent the S.D. and the points represent an average of at least three independent experiments.

Figure 2 shows the mouse Pgp-coupled ATPase activity of Pgp with verapamil and digoxin. In the absence of drugs, Pgp had basal ATPase activity of 512±151 nmol·min^−1^·mg^−1^ at saturating 3.2 mM ATP, which is in the range of basal activity rates observed in the literature between 0 [69] and 2600 nmol·min^−1^·mg^−1^ [70].

Kinetics of Pgp-coupled ATP hydrolysis in the presence of verapamil (Figure 2, open squares) was biphasic with substrate activation and inhibition of ATP hydrolysis reaching a maximum of 2106±98 nmol·min^−1^·mg^−1^ or 3- to 4-fold activation at 8 μM verapamil. Fitting the kinetics to the substrate inhibition equation (eqn 2) produced values for *V*_MAX_, *K*_m_ and *K*_i_ of 2546±130 nmol·min^−1^·mg^−1^, 1.9±0.5 μM and 454±109 μM respectively. These results suggest that there is a high-affinity and a low-affinity verapamil-binding site on Pgp.

Biphasic verapamil kinetics with the transporter has been observed previously with hamster [70–73], human [20] and mouse [74] Pgp. These values are very similar to the average *K*_m_ and *K*_i_ values determined for mouse Pgp in Ehrlich membranes of 2.5 and 225 μM [74] and for human Pgp in NIH-MDR1-G185 cells of 1.0 and 843.6 μM respectively [20]. The *V*_MAX_ was also similar to previous determinations with mouse Pgp and the half maximal ATPase activity of 4.2 μM with mouse Pgp was close to our estimates [39].

However, in a previous study [39], verapamil ATPase activation kinetics was monophasic and the maximum velocity was reached at 150 μM rather than 8 μM. Since the transporter is known to be sensitive to detergent and lipid composition [40,73,75–79], these differences in ATP hydrolysis kinetics were attributed to our procedure for reconstituting the transporter into the liposomes and our efforts to minimize the DDM during the protein purification process.

The digoxin-induced activation of ATP hydrolysis kinetics was monophasic and reached a maximum ∼2-fold activation or ∼1300 nmol·min^−1^·mg^−1^ (Figure 2, open circles), which is in the range observed previously [80–82]. The kinetics were fit to the Michaelis–Menten equation (eqn 1) and gave values for *V*_MAX_ and *K*_m_ of 1344±149.8 nmol·min^−1^·mg^−1^ and 240.4±68.1 μM respectively. This value is close to the *K*_m_ for digoxin transport in human Pgp from Caco-2 cells of 385 μM [83]. Although our *K*_m_ value was in the general range of previously determined *K*_m_ values for digoxin-induced ATPase activation of Pgp, the previously determined *K*_m_ values vary widely in the literature [80–82]. A *K*_m_ value of 1.2 μM for ATPase activation by digoxin was determined in Caco-2 membrane vesicles containing human Pgp [80], whereas a *K*_m_ value of 83.7 μM for ATPase activation was reported for human Pgp-enriched insect cell membranes [82]. For CR1R12 cells containing Pgp, maximal activation of ATP hydrolysis was not even reached at 1000 μM digoxin [83] implying a *K*_m_ that is considerably higher than 500 μM. This wide variation may be due to differences in membrane preparation, in lipid composition and/or in protein/lipid ratios.

### The effect of verapamil on the affinity of digoxin to Pgp by intrinsic protein fluorescence

Quenching of intrinsic protein fluorescence was used to probe the binding affinities of verapamil and digoxin to Pgp. Unfortunately, Pgp fluorescence at ∼330 nm was severely masked by inner filter effects and background fluorescence by verapamil when the protein was excited between 260 and 280 nm. Exciting the protein at 295 nm minimized these negative effects of verapamil on the protein fluorescence signal. Unlike previous studies with hamster Pgp [75], no significant verapamil-induced quenching of *F*_corrected_ was observed. However, this characteristic allowed us to examine the effects of verapamil on the affinity of digoxin to Pgp.

**Figure 3:**
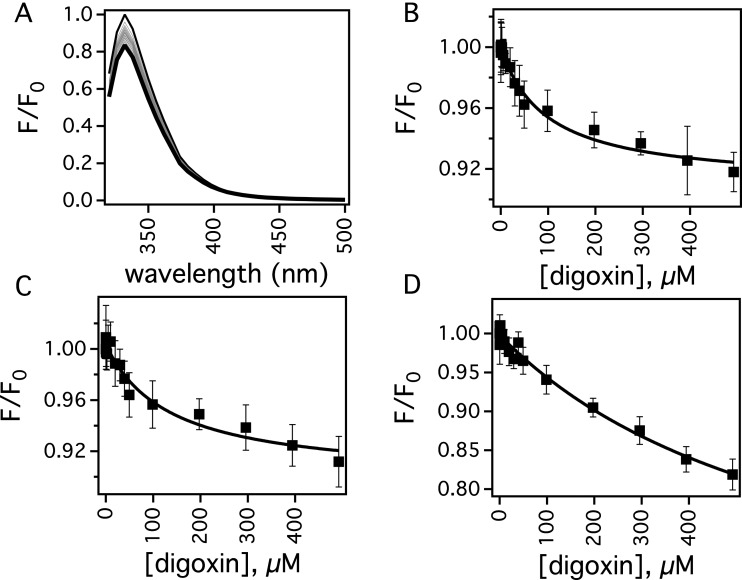
Digoxin-induced fluorescence quenching of Pgp in the presence of verapamil at 25°C (**A**) Pgp fluorescence spectra in the presence of a range of digoxin concentrations after exciting at 280 nm. The spectrum at 0 and 250 μM digoxin are shown as thin and thick lines, respectively, whereas intermediate concentrations of digoxin are shown as grey lines. Protein fluorescence emission at 333 nm as a function of digoxin concentration and in the presence of (**B**) 0 μM, (**C**) 8 μM and (**D**) 50 μM verapamil. The average and S.D. are represented as points and bars, respectively, and reflect at least three independent experiments.

Figure 3 shows the effect of digoxin on the protein fluorescence of Pgp in the presence of low and high concentrations of verapamil. Pgp was most sensitive to protein fluorescence quenching by digoxin when the protein was excited at 280 nm. Figure 3A shows the effect of a range of digoxin concentrations on the uncorrected normalized protein fluorescence of Pgp after exciting at 280 nm. After correcting the fluorescence with eqn (3), the amplitude at 333 nm in panel A was plotted as a function of the digoxin concentration in Figure 3B and shows that Pgp is quenched ∼10% at saturating levels of digoxin. The titration curve appears to be monophasic with a *K* of 0.0100±0.0018 μM^−1^ after fitting to eqn (4). To determine if the digoxin-induced quenching was due to a dynamic or a static quenching process, the titration was also performed at 37°C, which caused a decrease in the *K* value to 0.0030±0.0008 μM^−1^ and showed that digoxin induced static quenching of Pgp. This allowed us to calculate a dissociation constant (*K*_D_) for digoxin binding to Pgp at 25°C of 100±18 μM (i.e. *K*_A_=1/*K*_D_). A digoxin titration of Pgp was performed in the presence of 8 μM verapamil, which caused the highest activation of Pgp-coupled ATP-hydrolysis in Figure 2 (closed squares). The *F*_corrected_ at 333 nm was plotted as a function of protein concentration and is shown in Figure 3C. A *K*_A_ value of 0.0074±0.0033 μM^−1^ (*K*_D_=135±61 μM) was extracted from fitting the curve, which was very similar to the value determined in the absence of verapamil implying that both verapamil and digoxin are bound simultaneously to Pgp. The verapamil concentration was increased to 50 μM with the *F*_corrected_ digoxin titration shown in Figure 3D. The *K*_A_ determined by fitting this fluorescence quenching curve was 0.0015±0.00057 μM^−1^ (*K*_D_=679±261 μM). This is significantly lower than the *K*_A_ value determined at 8 μM and 0 μM verapamil. The decrease in *K*_A_ suggest that verapamil and digoxin are competitive at higher verapamil concentrations and that there is overlap in their binding sites. Digoxin titrations at higher verapamil concentrations with Pgp were attempted, but suffered from significant interference from inner filter effects by and fluorescence from verapamil (results not shown).

### Drug-induced conformational changes of Pgp by verapamil and digoxin

**Figure 4:**
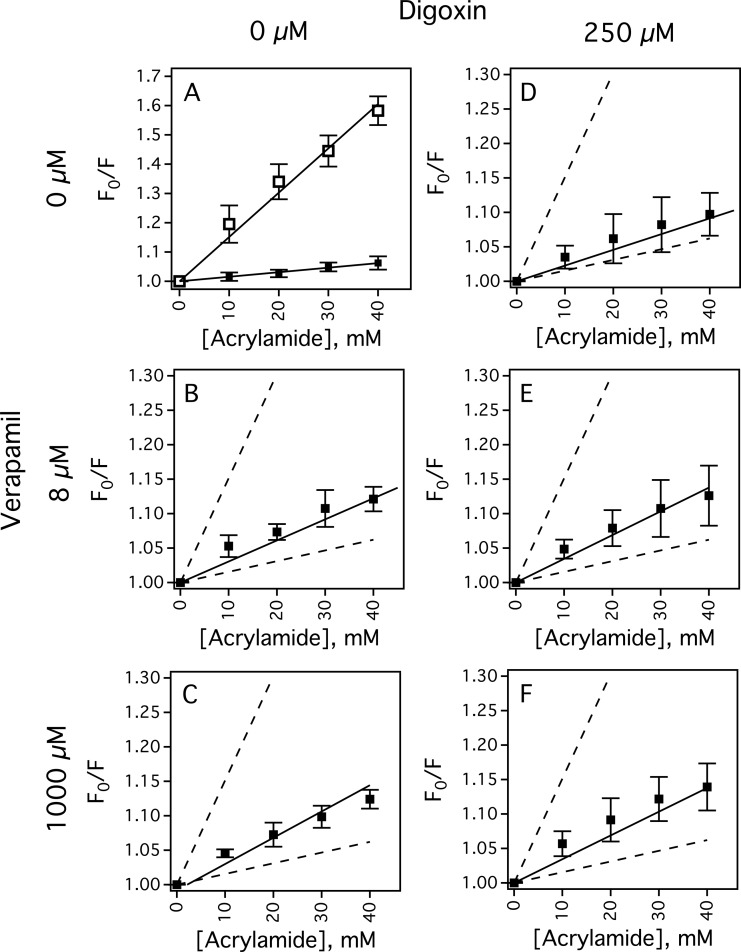
Acrylamide quenching of the Pgp transporter in the presence of verapamil and digoxin (**A**) The Stern–Volmer plots of NATA (open squares) and Pgp in the absence of drugs (closed squares). The Stern–Volmer plots of Pgp in the presence of (**B** and **E**) 8 μM and (**C** and **F**) 1000 μM verapamil. (**D**–**F**) The Stern–Volmer plots of Pgp with 250 μM digoxin added in addition to verapamil. For comparison, the slopes in panel (**A**) are presented as dashed lines in panels (**B**) through (**F**). The average and S.D. are represented as points and bars, respectively, and reflect at least three independent experiments.

Acrylamide quenching of tryptophan fluorescence in the presence of drugs was used to investigate drug-induced conformational changes of Pgp. Figure 4 shows Stern–Volmer plots (i.e. *F*_0_/*F* compared with [acrylamide]) in the absence and presence of drugs to probe protein conformational changes and tryptophan accessibility. The slope of the Stern–Volmer plot for Pgp in the absence of drugs had a *K*_SV_ value of 1.55±0.04 M^−1^ (Figure 4A, closed squares). The slope of the Stern–Volmer plot with NATA (Figure 4A, open squares) was measured to determine non-specific tryptophan interactions, and had a relatively high *K*_SV_ value of 15.14±0.57 M^−1^ that showed most of the tryptophans of Pgp are inaccessible to acrylamide. Figures 4B and 4C show the Stern–Volmer plots of Pgp in the presence of low and high concentrations of verapamil. *K*_SV_ values of 3.06±0.21 M^−1^ and 3.81±0.26 M^−1^ were determined from the slopes of the plots with low and high concentrations of verapamil respectively. These differences show that verapamil shifts Pgp into at least two distinct conformations and the largest conformational changes occur at low concentrations of verapamil. This observation is consistent with verapamil-induced Pgp conformational changes deduced from cross-linking of [84–86], trypsin digestion of [87] and antibody competition with Pgp [88]. The slope of the Stern–Volmer plot for Pgp in the presence of 250 μM digoxin was 2.29±0.12 M^−1^ (Figure 4D). When 8 μM of verapamil was added to 250 μM digoxin (Figure 4E), the *K*_SV_ value increased to 3.44±0.19 M^−1^. Addition of high concentrations of verapamil to Pgp in the presence of 250 μM digoxin increased the slope of the Stern–Volmer plot to 3.87±0.26 M^−1^ (Figure 4F), which is similar to the *K*_SV_ value determined from Figure 4C without digoxin and implies that they are in a similar conformation.

### Interactions of verapamil and digoxin with Pgp determined by STDD NMR

**Figure 5:**
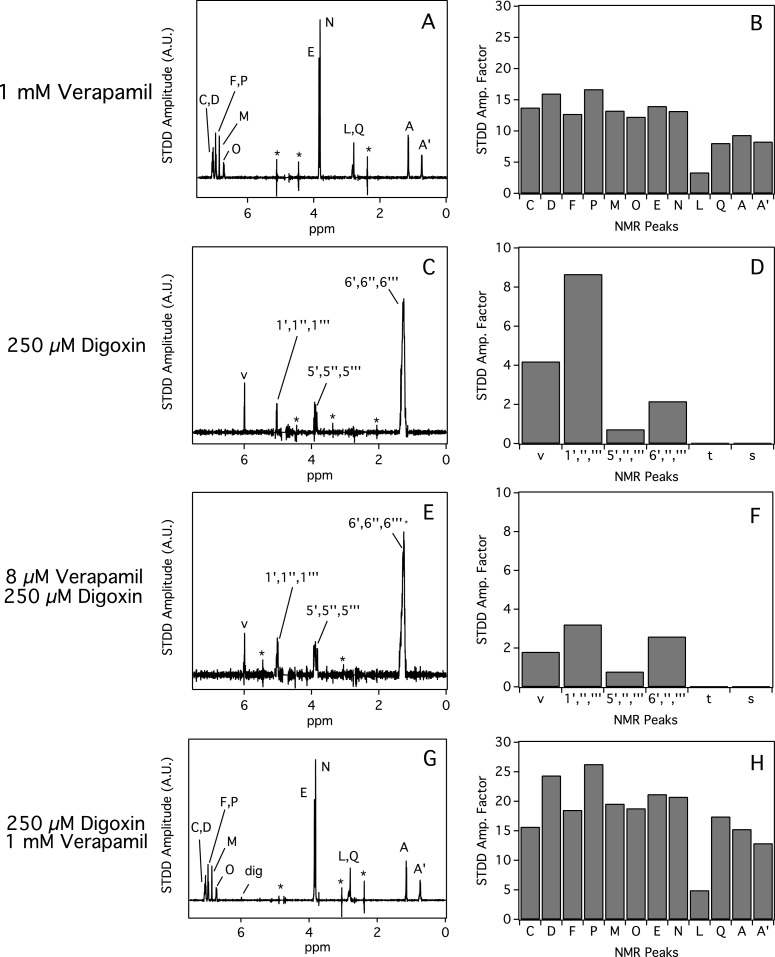
STDD NMR of verapamil and digoxin with 1 μM Pgp The STDD amplification (amp.) factors were calculated from the STDD NMR spectra (**A**, **C**, **E** and **G**) for verapamil (**B** and **H**) and digoxin (**D** and **F**). The concentrations of verapamil and digoxin are shown on the left side of the figure. Parameters for the NMR experiments are in the ‘Experimental’ section.

STDD NMR was used to probe the interactions of verapamil and digoxin with Pgp. Figure 5 shows the STDD NMR of verapamil and digoxin with Pgp. Figures 5A and 5B show the STDD NMR spectrum and amplification factors, respectively, with 1 mM verapamil and 1 μM Pgp. Overall, the strongest interactions with Pgp occurred with the aromatic and methoxy groups of verapamil with an STDD amplification factor of ∼15 indicating that they are the most important functional groups for molecular recognition by Pgp. STDD amplification factors that were half of these groups were observed for the methyls labelled A, A′ and Q with STDD amplification factors of ∼7. There were some weak STDD signals observed from the alkyl group (labelled L) of the distal phenyl group. No ^1^H STDD NMR peaks were observed for the other protons labelled G, H, I and K.

Figures 5C and 5D show the STDD NMR spectrum and amplification factors of 250 μM digoxin with Pgp. Significant STDD NMR peaks were observed for several protons (e.g. 1″′) emanating from the sugars and proton from the furan-2-one functional group. The highest STDD amplification factor was observed for the proton that is near the 1,4 β-linkage with an STDD amplification factor of ∼8. To investigate the effect of verapamil on the interactions of digoxin with the transporter, 8 μM of verapamil was added to samples containing protein and 250 μM digoxin in Figures 5E and 5F. Because of the low verapamil concentration, no STDD NMR peaks were observed for this drug. The relative amplitudes of the STDD NMR spectrum were quite similar to the STDD NMR spectrum taken without 8 μM verapamil. Therefore, low concentrations of verapamil did not significantly perturb digoxin's bound orientation to Pgp. However, the absolute amplitudes of the STDD NMR spectrum and amplification factors decreased ∼50% in the presence of 8 μM verapamil. This decrease was attributed to a fraction of verapamil molecules competing with digoxin bound to Pgp and to small errors in measuring the drug/protein ratios. The effect of higher concentrations of verapamil on digoxin's interaction with Pgp is shown in Figures 5G and 5H. No digoxin STDD NMR peaks were observed in the STDD NMR spectrum, which indicates complete displacement of digoxin from Pgp. The STDD amplification factors of verapamil were very similar to the ^1^H STDD NMR spectrum without digoxin (Figure 5A).

### Modelling Pgp-coupled ATPase activity with a panel of digoxin and verapamil concentrations

**Figure 6:**
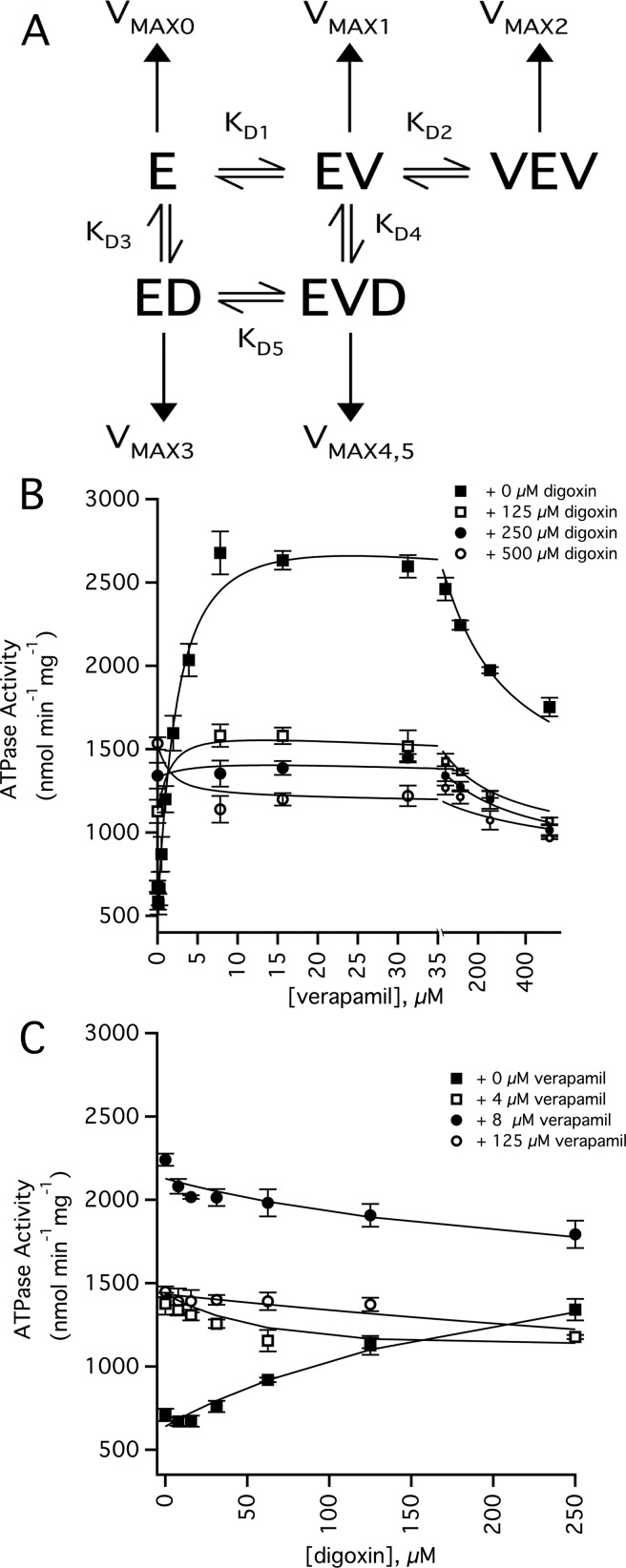
DDI effects of verapamil and digoxin on the ATPase activity of Pgp (**A**) DDI model used to fit the ATPase activity curves. Horizontal and vertical arrows denote the equilibria between bound states and the ATPase activity from the bound states respectively. E, V and D correspond to Pgp, verapamil and digoxin respectively. (**B**) Verapamil-induced activation of ATPase activity in the presence of 0 μM (closed squares), 125 μM (open squares), 250 μM (closed circles) and 500 μM digoxin (open circles). (**C**) Digoxin-induced activation of ATPase activity in the presence of 0 μM (closed squares), 4 μM (open squares), 8 μM (closed circles) and 125 μM verapamil (open circles). The fits are shown as lines, the error bars represent the S.D. and the points represent an average of at least three independent experiments. The statistics and the values used to fit the curves are shown in Supplementary Table S1 of the Supplementary Information.

Figure 6 shows a DDI model and Pgp-coupled ATPase activity curves with a panel of digoxin and verapamil concentrations. The model shown in Figure 6A was the simplest that encompassed the results of the ATPase activity, intrinsic tryptophan fluorescence and the STDD NMR experiments. In the model, two verapamil molecules bind to Pgp, which is consistent with the biphasic ATP hydrolysis kinetics shown in Figure 2. The model also shows that verapamil and digoxin bind simultaneously to Pgp (i.e. the enzyme verapamil-digoxin complex (EVD)). This is supported by the fact that the digoxin *K*_D_ is not significantly perturbed at low concentrations of verapamil and is also consistent with non-competitive inhibition for digoxin transport by verapamil [36,37]. In the model, higher concentrations of verapamil competitively displaces digoxin from its binding site on Pgp. Competitive displacement of digoxin by verapamil was observed at 50 μM verapamil in the intrinsic tryptophan measurements of Pgp (Figure 3D). It was also demonstrated in the STDD NMR spectrum in Figure 5G by a lack of ^1^H digoxin STDD NMR peaks.

Figures 6B and 6C shows the Pgp ATPase activity with a range of digoxin and verapamil concentrations. Because of the complexity of the model shown in Figure 6A, the kinetics curves in Figures 6B and 6C were fit using the COPASI software package. A complete list of kinetic and thermodynamic parameters used to fit the curves in the figures is presented in Supplementary Table S1 of the Supplementary Information. The fits to the ATPase activity kinetic curves had correlations (*R*) that were 0.9 or greater with one exception, which had a low χ^2^. The average basal ATP hydrolysis activity (*V*_MAX0_) determined from the fits was 538±64 nmol·min^−1^·mg^−1^.

Figure 6B shows the effect of digoxin on the ATPase activity with a range of verapamil concentrations. In the absence of digoxin, *K*_D1_, *K*_D2_, *V*_MAX1_ and *V*_MAX2_ values of 1.83 μM, 211 μM, 3000 nmol·min^−1^·mg^−1^ and 1100 nmol·min^−1^·mg^−1^, respectively, for verapamil-induced activation of Pgp-coupled ATP hydrolysis were extracted from the fits. These dissociation constants were very similar to those obtained by fitting the ATPase activity kinetics curve in Figure 2 (open squares). Fitting all of the curves gave an average *K*_D1_, *K*_D2_, *V*_MAX1_ and *V*_MAX2_ for verapamil-induced activation of Pgp-coupled ATP hydrolysis of 1.95±0.89 μM, 187±41 μM, 2757±313 nmol·min^−1^·mg^−1^ and 896±132 nmol·min^−1^·mg^−1^ respectively.

Figure 6C shows the effect of verapamil on the ATPase activity with a range of digoxin concentrations. In the absence of verapamil, the ATPase activity kinetics with digoxin was monophasic and fits well to the model in Figure 6A with a *K*_D3_ and *V*_MAX3_ of 239 μM and 1983 nmol·min^−1^·mg^−1^, which is similar to the *K*_D_ and *V*_MAX_ values obtained from fitting Figure 2 (open circles). The average *K*_D3_ and *V*_MAX3_ values for digoxin-induced ATPase activation determined from fitting all the curves with COPASI were 206±53 μM and 1981±207 nmol·min^−1^·mg^−1^.

The remaining parameters were estimated indirectly by fitting with COPASI. The affinity of digoxin to Pgp in the presence of verapamil (*K*_D4_) was 292±89 μM. This is very similar to the *K*_D2_ determined in the absence of verapamil. The affinity of verapamil to Pgp in the presence of digoxin (*K*_D5_) was 3.41±1.91 μM, which is relatively close to *K*_D1_. These results suggest that verapamil and digoxin were essentially not cooperative with respect to binding to Pgp. The *V*_MAX4,5_ for drug-induced ATPase activation from simultaneous binding of digoxin and verapamil was 121±139 nmol·min^−1^·mg^−1^ and reflects an almost complete inhibition of ATP hydrolysis in the presence of both drugs. In this case, verapamil and digoxin are negatively cooperative with respect to Pgp-coupled ATP hydrolysis. This also correlates well with non-competitive inhibition of digoxin transport by Pgp in the presence of verapamil [36,37].

## DISCUSSION

**Figure 7:**
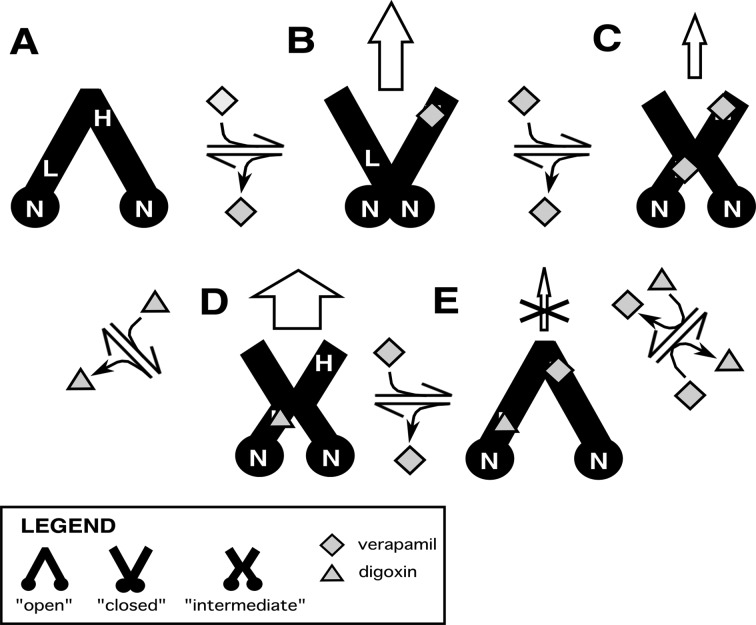
DDI transport model of verapamil and digoxin with Pgp Pgp is shown as a cartoon representation of three conformational states: ‘open’, ‘closed’ and ‘intermediate’. Verapamil and digoxin are represented as diamonds and triangles respectively. The panels show Pgp (**A**) in the absence of drugs, (**B**) with 1 bound verapamil molecule, (**C**) with 2 bound verapamil molecules, (**D**) with 1 digoxin molecule bound and (**E**) with 1 bound verapamil and 1 bound digoxin. The top and the bottom of the Pgp representations are the extracellular and cytosolic sides respectively. The vertical arrows denote transport and the size of the arrows reflect their relative transport rates, whereas X denotes transport inhibition. H, L and N are the high-affinity binding site, low-affinity binding site and the NBDs respectively.

In Figure 7, we propose a DDI transport model with Pgp based on our results with verapamil and digoxin, and the conformational changes that Pgp is known to undergo with nucleotide cofactors and drugs [85,86,89,90]. For simplicity, we have represented Pgp in our model by three conformations: ‘open’, ‘closed’ and ‘intermediate’. In reality, these conformations represent an ensemble average between a range of conformations. In the ‘open’ conformation, the NBDs are relatively far apart and the cytosolic side is exposed to the bulk solvent. In the ‘closed’ conformation, the NBDs are in contact with each other and the extracellular side is exposed to the bulk solvent. The ‘intermediate’ conformation is between the ‘open’ and ‘closed’ conformations. In this conformation, both the cytosolic and extracellular sides of Pgp are exposed to the bulk solvent.

Drug-induced changes in tryptophan accessibility deduced from the acrylamide quenching experiments implied that Pgp occupies distinct conformations at each of the digoxin and verapamil concentrations. Unfortunately, this information cannot be used to assign specific drug-bound Pgp conformations. Instead, the assignment was based on drug-induced activation of the Pgp-coupled ATP hydrolysis rate. Our rational was based on the fact that site-directed mutagenesis and cross-linking studies of Pgp in addition to structural studies of the bacterial transporters with nucleotide analogues have demonstrated that the interaction of the Pgp nucleotide domains with each other is essential for ATP hydrolysis [86,91–96]. Therefore, the average distance between the NBDs of Pgp was correlated to the ATP hydrolysis rate in our model. In other words, drugs that induce a relatively low and high ATPase rates will shift Pgp into ‘open’ and ‘closed’ conformations respectively.

The locations of the verapamil-binding sites are currently unknown. The biphasic verapamil ATPase activation kinetics that are shown in Figures 2 and 6 suggest a high- and a low-affinity verapamil-binding site on Pgp. Several studies have identified residues clustered near the extracellular side of Pgp [97–99] and G185 [100,101], which lies in the transmembrane region of Pgp, that have marked effects on verapamil-induced activation of ATP hydrolysis and transport. Deletion of residues between 78 and 97 near the extracellular side of human Pgp caused a dramatic increase in the *K*_m_ for ATPase activation by verapamil [97]. Multiple mutations near the extracellular side of human Pgp decreased activity towards verapamil transport [99]. Permanent ATPase activation of human Pgp was observed in cysteineless human Pgp with an I306C mutation labelled with a thiol-reactive verapamil analogue [98]. Mutating the G185 residue had very strong effects on the *V*_MAX_ of verapamil-induced ATPase activation [100,101]. The mutation also had significant effects on the *K*_i_ for substrate inhibition for verapamil, but negligible effects on verapamil's *K*_m_ [100]. By affecting the *K*_i_ and not the *K*_m_ suggested to us that the mutation is affecting an alternate verapamil-binding site. With this information, the high-affinity (H) drug-binding site is placed roughly near the extracellular side of Pgp, whereas the low-affinity (L) drug-binding site is closer to the NBDs within the transmembrane region of the transporter in Figure 7A.

Figure 7A shows Pgp in the absence of ligands. Because the ATPase hydrolysis rate in the absence of ligands is relatively low at ∼500 nmol·min^−1^·mg^−1^, Pgp will be in an ‘open’ conformation with the NBDs (N) separated in our model.

At low verapamil concentrations, the drug binds to the H site in Figure 7B. Fitting the Pgp-coupled ATPase activity kinetics of Figure 6B gave a *V*_MAX_ of ∼3000 nmol·min^−1^·mg^−1^. This is the highest ATPase activation observed for either drug. Therefore, Pgp is proposed to be in the ‘closed’ conformation under these conditions.

At higher verapamil concentrations, the drug will occupy the L site on Pgp in Figure 7C. The degree of ATPase activation is less than half the Pgp-coupled ATPase activation at lower verapamil concentrations, but is significantly higher than basal Pgp-coupled ATPase activity. Therefore, Pgp is proposed to be in an intermediate conformation. Consistent with the concentration-dependence observed for ATPase activation by verapamil, the drug transport rate is also concentration-dependent. In Caco-2 cells containing human Pgp, verapamil had a higher permeability ratio with Pgp at low opposed to higher verapamil concentrations [23]. Also, human Pgp overexpressed in LLC-PK1 cells had higher efflux ratios at 350 nM than 5 μM verapamil [21,25]. Therefore, we propose that verapamil occupancy at the H site alone (Figure 7B) will lead to higher verapamil transport rates than occupancy at both drug-binding sites (Figure 7C).

Addition of digoxin leads to formation of the Pgp complex shown in Figure 7D. The affinities deduced from the intrinsic protein fluorescence (Figure 3) and from fitting the ATPase activity kinetics curves (Figures 2 and 6) posits the drug in the L site. The degree of Pgp-coupled ATPase activation by digoxin was similar to the Pgp-coupled ATPase activation in the presence of high concentrations of verapamil. The relative tryptophan accessibility determined from the slopes of the Stern–Volmer plots was similar under both of these conditions (cf. Figures 4E and 4C). Therefore, Pgp will be in an intermediate conformation in our model. The permeability/efflux ratios of digoxin with Pgp in several cell lines ranged between 4 and 35 [21,24,102,103]. This contrasts with the permeability/efflux ratios for Pgp at low verapamil concentrations, which were generally lower and ranged from ∼1 to 6 [21,23,24]. These results suggest that the coupling between ATP hydrolysis and transport for drugs may be ligand dependent.

When low verapamil concentrations are added to the digoxin–Pgp complex, verapamil will occupy the H site and form the complex shown in Figure 7E. Several lines of evidence support the simultaneous binding of verapamil and digoxin to Pgp. First, verapamil non-competitively inhibits digoxin transport by Pgp [36,37]. Second, the *K*_D_s determined from fitting the intrinsic protein fluorescence quenching curves in Figure 3 showed that addition of low concentrations of verapamil does not significantly change the *K*_D_ of digoxin to Pgp. Third, there are significant ^1^H STDD NMR peaks for digoxin at 8 μM verapamil (Figure 5E), which is a high enough verapamil concentration to saturate the H site. Fitting the Pgp-coupled ATPase activity curves in Figure 6 revealed that binding of both drugs will inhibit ATP hydrolysis. Therefore, Pgp will be in the ‘open’ conformation.

Higher concentrations of verapamil will completely displace digoxin from the L site forming the double bound complex in Figure 7C. This configuration is supported by our results that showed the affinity decreased significantly at verapamil concentrations above 8 μM (Figure 3). This is also supported by the complete loss of ^1^H STDD NMR signals from digoxin in the presence of 1 mM verapamil (Figure 5G) and implied by the similarity of the Stern–Volmer plots of Pgp with 1 mM verapamil in the absence and presence of 250 μM digoxin (cf. Figures 4C and 4F).

## Abbreviations

COPASI: complex pathway simulator
DDI: drug–drug interaction
DDM: *n*-dodecyl-β-D-maltoside
EGTA: ethylene glycol tetraacetic acid
*L*: ligand
NATA: *N*-acetyl-L-tryptophanamide
NBD: nucleotide-binding domain
Ni-NTA: nickel-nitrilotriacetic acid
Pgp: P-glycoprotein
STD: saturation transfer difference
STDD: saturation transfer double difference
